# Identification of a protective B-cell epitope of the *Staphylococcus aureus* GapC protein by screening a phage-displayed random peptide library

**DOI:** 10.1371/journal.pone.0190452

**Published:** 2018-01-05

**Authors:** Mengyao Wang, Lu Zhai, Wei Yu, Yuhua Wei, Lizi Wang, Shuo Liu, Wanyu Li, Xiaoting Li, Simiao Yu, Xiaoting Chen, Hua Zhang, Jing Chen, Zhenyue Feng, Liquan Yu, Yudong Cui

**Affiliations:** 1 College of Life Science and Technology, Heilongjiang Bayi Agricultural University, Daqing, P.R. China; 2 College of Animal Science and Technology, Heilongjiang Bayi Agricultural University, Daqing, P.R. China; Instituto Butantan, BRAZIL

## Abstract

The impact of epidemic *Staphylococcus aureus* (*S*. *aureus*) on public health is increasing. Because of the abuse of antibiotics, the antibiotic resistance of *S*. *aureus* is increasing. Thus, there is an urgent need to develop new immunotherapies and immunoprophylaxes. Previous studies showed that the GapC protein of *S*. *aureus*, which is a surface protein with high glyceraldehyde 3-phosphate dehydrogenase activity, transferrin binding activity, and other biological activities, is highly conserved. GapC induces an effective humoral immune response *in vivo*. However, the B-cell epitopes of *S*. *aureus* GapC have not been well identified. Here we used the bioinformatics tools to analyze the sequence of GapC, and we generated protective anti-GapC monoclonal antibodies (mAbs). A protective mAb (1F4) showed strong specificity to GapC and the ability to induce macrophages to phagocytose *S*. *aureus*. We screened the motif ^272^GYTEDEIVSSD^282^, which was recognized by mAb 1F4, using a phage display system. Then, we used site-directed mutagenesis to identify key amino acids in the motif. Residues G272 D276 E277 I278 and V279 formed the core of the ^272^GYTEDEIVSSD^282^ motif. In addition, we showed that this epitope peptide induced a protective humoral immune response against *S*. *aureus* infection in immunized mice. Our results will be useful for the further study of epitope-based vaccines against *S*. *aureus* infection.

## Introduction

*Staphylococcus aureus*, one of the most important pathogens, causes human soft tissue suppurative infections, pneumonia, endocarditis, osteomyelitis, and septicemia [1]. It is also one of the major pathogens associated with human-related medical devices and artificial joint infections [2, 3]. In animals, it also causes a variety of infections, such as bovine mastitis, chicken suppurative arthritis, and pig abortions, which have been causing huge economic losses to the animal husbandry industry. Over the years, there has been a committment to prevent the *S*. *aureus* infections, and antibiotic therapy has always been the main method, but the excessive use and abuse of antibiotics has led to the increasing emergence of resistant strains. Previous studies confirmed that many *S*. *aureus* strains are resistance to every clinical antibiotic, such as vancomycin [4, 5], linezolid [6, 7], daptomycin [8] and mupirocin [9]. Therefore, developing new immunotherapies, particularly vaccine control approaches, is being investigated extensively. One strategy is to develop effective monoclonal antibodies (mAbs) and vaccines against *S*. *aureus* [10, 11].

*Staphylococcus aureus* expresses a series of surface proteins that regulate the binding of bacteria to plasma proteins and extracellular matrix components, thereby promoting the colonization and release of toxins at different locations *in vivo* [12]. Additionally, some of these surface proteins, such as IsdB, SasA and ClfA [13–15], have been tested as candidate targets for vaccines against *S*. *aureus*. One of these surface proteins, glyceraldehyde-3-phosphate dehydrogenase C (GapC), possesses GAPDH activity and reversibly catalyzes the conversion of glyceraldehyde-3-phosphate into 1,3-bisphosphoglycerate by phosphorylation. GAPDH is a stimulatory protein that induces the proliferation and differentiation of B cells by inducing intereukin-10 production [16]. Previous studies demonstrated that the *S*. *aureus* GapC protein plays a role in the adherence to and internalization into bovine mammary epithelial (MAC-T) cells [17]. Meanwhile, GapC induces an effective humoral immune response and immunoprotection against *S*. *aureus* infections, which suggests that GapC might be a good immunodominant antigen [18–20].

As is well known, B-cell immunity provides a natural barrier for a host to block the invasion of pathogens into its cells mainly through B-cell epitope, which are peptide fragments of an antigen that can be recognized and trigger a B-cell immune response [21]. B-cell epitopes are the key for inducing a humoral immune response [22]. B-cell epitopes generally contain 5–7 amino acids, and no more than 20 amino acids. However, the B-cell epitopes on the *S*. *aureus* GapC protein have not been well characterized until now. In this study, we generated a mAb, 1F4, that targets GapC. The B-cell epitope recognized by mAb 1F4 was screened, and its core amino acids were analyzed. Animal experiments proved that the GapC epitope peptide recognized by mAb 1F4 induced a protective humoral immune response against *S*. *aureus*.

## Materials and methods

### Mice, ethics statement and production of anti-GapC mouse serum

Specific-pathogen-free BALB/c mice (female, 6–8 weeks) were provided by the Changchun Institute of Biological Products (Changchun, China). The mice were supplied with water and food *ad libitum* and allowed to acclimatize for a period of 7 days before the corresponding experiment. The humane endpoints were used for all animals involved in the study. Care of laboratory animals and animal experimentation were performed in accordance with animal ethics guidelines and approved by the Animal Ethics Committee of HeiLongJiang BaYi Agricultural University. In the survival study, we used humane endpoints and euthanized mice displaying severe illness prior to the end of our experiments to minimize pain and distress. The criteria for determining when the animals should be euthanized included weight loss, appetite loss, weakness, lack of feeding or drinking, signs of severe organ system dysfunction, ruffled fur, non-responsive to treatment. At the completion of all animal experiments, survivors were euthanized by CO_2_ exposure with IACUC policy. Death was verified by monitoring cardiac cessation and respiratory arrest. All efforts were made to minimize suffering. The anti-GapC mouse serum was prepared in our lab. BALB/c mice were immunized subcutaneously with 50 μg of GapC emulsified with Freund’s adjuvant. Then, two boosts were immunized with incomplete Freund’s adjuvant at the same dose for 2-week at each interval. Two weeks after the final boost, the BALB/c mice were anesthetized, humanely sacrificed and blood. The anti-GapC mouse serum was isolated from coagulated blood, and the concentration of anti-GapC immunoglobulins in this serum was determined about 100 μg/ml according to our previous study [23].

### Bacterial strains, plasmids and cell lines

*Staphylococcus aureus* strain Newman, a capsular type 5 strain, was obtained from the Eijkman Winkler Laboratory of the University Medical Center, Utrecht, the Netherlands. *S*. *aureus* were grown in tryptic soy broth or agar (Difco, Becton-Dickinson, Sparks, MD, USA) at 37°C. The *S*. *aureus gapC* gene was cloned into the pET-32a (+) plasmid and expressed in BL21 (DE3) (Novagen, Madison, WI, USA). The mutated versions of the epitope motifs were cloned into pGEX-6p-1 plasmid and expressed in BL21 (DE3). *Escherichia coli* DH5a (Invitrogen, Carlsbad, CA, USA) was used for cloning purposes. *E*. *coli* strains DH5α and BL21 carrying plasmids were grown in Luria-Bertani broth or agar (Difco) at 37°C, in the presence of an antibiotic (100 μg of ampicillin/mL) when necessary. The myeloma cell line SP2/0 was maintained in Roswell Park Memorial Institute 1640 medium supplemented with 10% fetal bovine serum (HyClone, USA) and 100 μg/ml penicillin/streptavidin. All cells were maintained at 37°C in 5% CO_2_.

### Preparation of recombinant GapC

The full-length *gapC* gene was amplified by polymerase chain reaction (PCR). The primers used in this study are listed in Table 1. The overlapping PCR conditions were as follows: an initial denaturation step at 94°C for 3 min, followed by 35 cycles of 94°C for 30 s, 59°C for 30 s, and 72°C for 1 min, followed by a 10-min incubation at 72°C and a final incubation at 4°C for 1 h. The PCR products were cloned into the pET-32a (+) vector and transformed into *E*. *coli* strain BL21 (DE3). Recombinant GapC with a six-histidine tag was expressed as described previously [24] and purified with a histidine—affinity chromatography purification system (CTB, China) following the manufacturer’s recommendations.

**Table 1:** Primers used in this study.

Primer	Sequence (5’–3’)	Description
G1	*CGCGGATCC*ATGGCAGTAAAAGTAGCAA	The forward primer of GapC, plus *Bam* HI site
G2	*CCCAAGCTT*TTTAGAAAGTTCAGCTAAG	The reverse primer of GapC, plus *Hind* III site

Note: *Bam*H I and *Hind* III restriction enzyme sites were introduced at the 5’ end of the primers as indicated italics.

### Antibody generation and purification

Murine mAbs targeting GapC were generated by the standard hybridoma method described in earlier studies [25, 26]. mAbs were generated in female, 12-week-old BALB/c mice. After three immunizations, the antibody titer of the mice serum was tested. Mice with a titer of 1:10,000 or greater were selected for the cell fusion experiment. Splenocytes were fused with SP2/0 myeloma cells and selected with hypoxanthine-aminopterin-thymidine (HAT) medium [27]. The culture supernatant from the hybridomas was screened by an enzyme-linked immunosorbent assay (ELISA). The mAb-producing hybridomas were cloned by limited dilution of the cells. After four rounds of screening, strongly positive hybridoma cell lines capable of stably secreting the antibodies were screened. The hybridomas (2×10^6^ cells/mouse) were injected intraperitoneally into mice, and after the mice exhibited a swollen abdomen, the ascites fluid was extracted with a syringe. mAb 1F4 was purified using a HiTrap^TM^ Protein G HP column (GE, USA) according to the manufacturer’s recommendations.

### Establishment of an ELISA

The optimal working concentrations of the coated antigen and serum were determined by titrations. The purified GapC protein was diluted 1:100, 1:200, 1:400, 1:800, 1:1,600, 1:3,200, 1:6,400, and 1:12,800 with 0.05 M carbonate buffer (pH 9.6), and 100 μl (per well) of each dilution was coated onto microplates (96-well) at 4°C. The liquid in the wells was discarded and then the wells were washed three or four times with phosphate-buffered saline containing 0.5% Tween-20 (PBST). The plates were blocked with 5% nonfat milk for 1 h at 37°C. The serum was diluted 1:100, 1:200, 1:400, 1:1,600, 1:3,200, and 1:6,400 with PBS and added to the wells. After washing, secondary horseradish peroxidase (HRP)-conjugated goat anti-mouse IgG (Origene, USA) was added and incubated at 37°C for 1 h. After washing, 3,3’,5,5’-tetramethylbenzidine dihydrochloride (Sigma, USA) was added as the substrate. The reaction was terminated with 2 M H_2_SO_4_, and the optical density at 450 nm (*OD*_450_) was determined using a microplate reader.

### Western blot analysis

Recombinant GapC was subjected to 12% sodium dodecyl sulfate-polyacrylamide gel electrophoresis (12% SDS-PAGE). The gel was electrotransferred onto a nitrocellulose membrane (NEB, USA) by western blotting at 115 mA for 90 min at room temperature. The membranes were blocked in blocking buffer for 90 min. After washing three times with PBST (0.5% Tween-20), the membranes were incubated with primary antibodies for 1 h at room temperature. The membrances were washed three times with PBST and incubated with HRP-conjugated goat anti-mouse IgG (Origene) at a 1:5,000 dilution for 1 h. The reaction was detected by a western HRP substrate.

### Characterization of mAbs

Hybridoma supernatants and ascites titers were determined by an indirect ELISA. 96-well plates were coated overnight with 1 μg/ml of purified recombinant GapC. After washing three times and blocking with 5% skim milk dissolved in PBS for 1 h at 37°C, 100 μl of the hybridoma cells’ supernatant or ascites fluid were added to the wells and incubated at 37°C for 1 h after washing. Then, the wells were washed three times with PBST, and then incubated with HRP-conjugated goat anti-mouse IgG at 37°C for 1 h. Estimation of enzyme activity was performed using the 3,3’,5,5’-tetramethylbenzidine dihydrochloride (TMB) substrate. The reaction was terminated with 2 M H_2_SO_4_, and the *OD*_450_ of each well was read using a microplate reader at 450 nm.

The subclasses and specificity of mAb 1F4 were detected by the same method. The mouse mAb Isotyping Kit (GE, UK) was used to detect subclasses, including IgM, IgG1, IgG2a, IgG2, IgG3, IgA, κ chains, and λ chains. The specificity of mAb 1F4 was tested, and the primary antibodies included those recognizing the *S*. *aureus* proteins, include IsdB [28], Trap [29], FnbpA [30], and Mntc proteins [31] and the *S*. *dysgalactiae* GapC protein [32].

The effect of mAb 1F4 on the GapC GAPDH activity of *S*. *aureus* was determined. The GAPDH activity of recombinant GapC was determined by a GAPDH activity assay kit (OMIN, China). The purified GapC was incubated with glyceraldehyde 3-phosphate and NAD^+^ in a final volume of 1 mL of assay buffer (triethanolamine, Na_2_HPO_4_, and EDTA). mAb 1F4 was incubated with the mixture at 37°C for 5 min, the decrease of NADH reflected the level of GAPDH activity by a spectrophotometer at *OD*_340_ after incubation.

### Screening a random phage-displayed 12 peptide library

According to previous studies, a random PhD-12 phage display peptide library (NEB, Beverly, MA, USA) was screened with the mAbs [33]. 96-well plates were coated with 50 μg/ml of the mAbs (100 μl/well) for 12 h at 4°C. After washing six times with Tris-buffered saline containing 0.1% (v/v) Tween-20 (TBST), 300 μl of blocking buffer was added and incubated at 4°C for 1 h. The diluted phage (1×10^11^) were incubated at room temperature with the bound mAb for 1 h and shaken slightly. After washing 10 times with TBST, 100 μl of the same mAbs at a concentration of 200 μg/ml were added and incubated with shaking at room temperature for 1h for competitive elution of the bound phages. The phage titer was determined by assaying 1 μl of the phage after elution. The remaining eluate was amplified by infecting 20 ml of *E*. *coli* strain ER2738. The culture was shaken vigorously at 37°C for 4.5 h. Finally, the supernatant was collected with polyethylene glycol/NaCl. The phage yield was calculated as: recovery = (eluted phage/input phage) × 100%. After three rounds of biopanning (the concentration of the Tween-20 in the TBST was increased in each round), positive phage clones were screened, and their reactivity with mAb 1F4 was verified by a sandwich ELISA. The single-stranded phage DNAs of the positive phage clones were extracted and sequenced with the primer 5'–TGAGCGGATAACAATTTCAC–3' and analyzed using DNASTAR software.

### Site-directed mutagenesis assay

To determine the key amino acids of the epitope, we replaced each amino acid residue of the epitopes with alanine. The wild-type and mutated versions of the epitope motifs were synthesized by Comate Bioscience Co.,Ltd and ligated them into the pGEX-6p-1 vector to produce recombinant plasmids. The recombinant plasmids were expressed in BL21 (DE3) to express the fusion protein with a glutathione S-transferase (GST) tag. Using mAb 1F4 as the primary antibody, key amino acids were identified by detecting of the reactivity of various fusion proteins with mAb 1F4 by western blotting.

### Confocal laser-scanning microscopic analysis

*Staphylococcus aureus* (Newman) was grown at 37°C for 4 h, and then 1 ml of the culture was removed and centrifuged at 12,000 × *g* for 1 min. The supernatant was discarded, and the sediment was washed twice with sterile PBS and then resuspended with 2 ml of PBS. Then, 20 μl of the bacteria was removed, spotted onto the microslides (soaked overnight in 75% ethanol), air-dried. Then, the slides were transferred into the cold acetone for 30 min. After washing the slides for 15 min with PBST, the slides were blocked with 5% skimmed milk at room temperature for 2 h and then washed with PBST for 30 min. After drying, 1:200 diluted anti-GapC serum was added, and mAb 1F4 (1 mg/ml) and SP2/0 (negative control) were used as the primary antibodies and placed at 4°C for 8 h. After washing 30 min with PBST, the slides were incubated with a fluorescein isothiocyanate-conjugated (FITC) secondary goat anti-mouse antibody (Bioss, China) (1:200 dilution) for 5 h at 4°C. Then, the slides were rinsed with water and covered with buffer (glycerol/PBS = 3/1). A LeicaTCS-SP8 confocal laser-scanning microscope was used to examination.

### Opsonocytophagic assay

The mice were humanely sacrificed, the peritoneal macrophages were harvested under aseptic conditions, and the macrophagocyte cell concentration was adjusted to 1×10^5^ cells/ml using Dulbecco’s modified Eagle’s medium (DMEM) containing 10% fetal bovine serum in a 24-well plates and incubated overnight at 37°C in a 5% CO_2_ (v/v). DMEM-washed *S*. *aureus* Newman strain was adjusted to a concentration of 2×10^5^ colony-forming units (CFU)/ml, resulting in the multiplicity of infection (MOI) of 1–10 [17, 34]. 50 μl of serum was added, and the mixture was incubated at 37°C (5% CO_2_) for 30 min. Then, the mixture was transferred to the 24-well plates with macrophages at 37°C for 1 h. After incubation, wells were washed 3 times with warm DMEM and extracellular adherent bacteria were killed by adding 0.5 mL of DMEM supplemented with gentamicin (100 μg/mL) and penicillin (5 μg/mL) for 2h [34, 35]. After incubation, the cellular supernatant was discarded, and the cells were washed 3 times with Hank’s balanced salt solution (HyClone). The cells were lysed with cold water at 4°C for 20 min. Viable internalized Newman were counted by plating serial dilutions on tryptic soy agar (in triplicate) of cell lysate and determining the number of CFUs.

### Mice immunization and challenge

For the positive immunization assay, female, 6–8-week-old mice were divided into four groups (*n* = 10). The dosage of each immunogen was 100 μg. The mice were further immunized two more times every 2 weeks. Freund’s adjuvant was used for the first immunization, and incomplete Freund’s adjuvant was used for the second and third immunizations. Two weeks after the third immunization, the mice were challenged intraperitoneally with 5×10^8^ CFU/mouse of *S*. *aureus*. Survival was monitored at daily for 15 days after the challenge.

For the passive immunization assay, female, 6–8-week-old mice were passively immunized intravenously with 200 μg of purified mAb 1F4 or SP2/0 cell supernatant (*n* = 10). Twenty-four hours later, the mice were challenged intraperitoneally with 5×10^8^ CFU/mouse of *S*. *aureus* (Newman). Survival was monitored for 7 days after the challenge. All mice were used in accordance with guidelines from the Committee of the Heilongjiang Bayi Agricultural University.

### Tertiary structure of the epitope

The PDB (http://www.rcsb.org/pdb/home/home.do) method and the PyMOL tool were used to analyze the tertiary structure of GapC and the epitope. The tertiary structure of Glyceraldehyde-3-phosphate dehydrogenase 1 from *S*. *aureus* MRSA252 was used as template [36] and the homology between the template and GapC from *S*. *aureus* Newman strain was 100%.

### Statistical analysis

All experiments were performed at least twice. Experimental data were analyzed with the Student’s *t*-test. The main statistical analyses were the opsonophagocytosis of *S*. *aureus* and the protection studies. Statistical significance was marked as ‘*’ when *p* < 0.05, ‘**’ when *p* < 0.01 and ‘***’ when *p* < 0.001. *p* <0.01 was considered to be statistically significant. Values are expressed as means ± standard deviations.

## Results

### Construction and expression of recombinant GapC

The 1,008-bp *gapC* gene was obtained by PCR amplification from the *S*. *aureus* Newman strain, cloned into the pMD18-T vector, identified by BamHI and HindIII digestion, and sequenced by Beijing Jin Wei Zhi Biological Technology Co., Ltd. The positive clones were compared with the *gapC* sequence in GenBank, and the results showed that they were 100% identical. To construct a GapC expression vector, the recombinant plasmid pMD18-T-GapC was digested with BamHI and HindIII, and the *gapC* gene was cloned into the pET-32a (+) plasmid. The recombinant plasmid pET-32a (+)-GapC was transformed into *E*. *coli* BL21 (DE3) competent cells. The recombinant plasmids were analyzed by PCR and double digestion with BamHI and HindIII (S1 Fig). The sequencing results demonstrated that the *gapC* sequence was identical to the known sequence.

**Fig 1:**
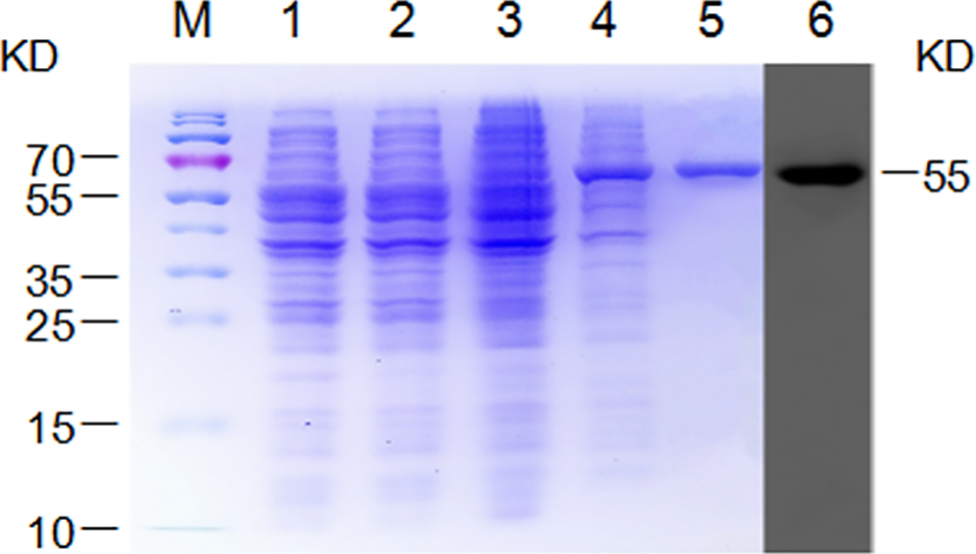
Expression and purification of recombinant GapC. The GapC fusion protein was detected by SDS-PAGE and western blotting using an anti-His antibody. Lane M, protein marker; Lane 1, *E*. *coli* BL21 with pET32a (+) prior to isopropyl β-D-1-thiogalactopyranoside (IPTG) induction; Lane 2, *E*. *coli* BL21 with pET32a (+) after IPTG induction for 4 h; Lane 3, *E*. *coli* BL21 with pET32a (+)/GapC prior to IPTG induction; Lane 4, *E*. *coli* BL21 with pET32a (+)/GapC after IPTG induction for 4 h; Lane 5, purified GapC protein; Lane 6, the GapC fusion protein was confirmed by western blotting using anti-His antibody.

Recombinant GapC expression was induced by IPTG and GapC was purified by histidine-affinity chromatography (CTB). SDS-PAGE showed that the molecular weight of recombinant GapC was approximately 55KDa, which is in accordance with the predicted molecular weight of GapC. Moreover, recombinant GapC was further confirmed by western blotting using an anti-His-tag antibody (Fig 1). The results showed that recombinant GapC was expressed correctly.

### Monoclonal antibodies to GapC

**Fig 2:**
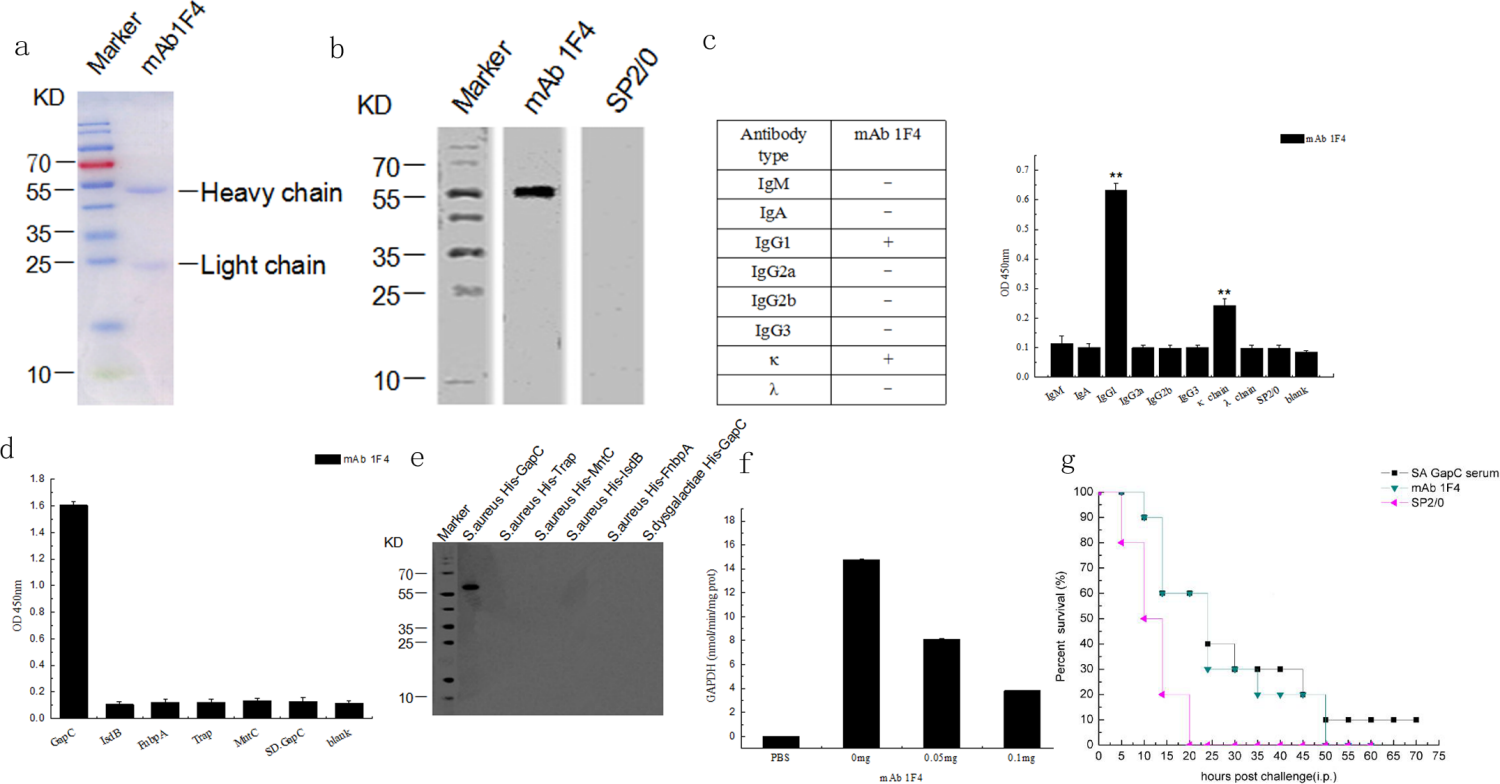
Characterization of mAbs. (**a**) The purified 1F4 was analyzed by 12% SDS-PAGE. (**b**) GapC recognized by the mAb 1F4 was detected by western blotting. (**c**) The class of the mAb 1F4 was determined to be IgG1 and the κ chain. (**d**) The specificity of mAb 1F4 toward recombinant FnbpA,Trap, Mntc, IsdB of *S*. *aureus* and GapC from *S*. *dysgalactiae* was determined by indirect ELISA. (**e**) The reactivity of mAb 1F4 with the recombinant FnbpA, Trap, Mntc, IsdB proteins of *S*. *aureus* and GapC from *S*. *dysgalactiae* was determined by western blotting. (**f**) Effect of 1F4 on GapC GAPDH activity of *S*. *aureus*. The OD values were detected at 20 s (A1) and 5 min and 20 s (A2), the decrease of NADH reflected the level of GAPDH activity at *OD*_340_. The data represent the means ± SEM (*n* = 3). (**g**) Passive immunization with mAb 1F4 protects against *S*. *aureus* infection. Mice were injected intraperitoneally with 5×10^8^ CFU of *S*. *aureus* (Newman) followed by the intravenous injection of anti-GapC serum, mAb 1F4, or SP2/0 cell supernatant (nagative control) after 24 h (*n* = 10).

According to the ELISA, the optimal concentration of the purified GapC protein and the positive serum was 1:1,600, as it resulted in the highest P/N value. We generated six hybridoma cell lines. mAbs were screened using ELISA assays according to the optimal dilution concentration. One of the cell lines stably secreted anti-GapC antibody at a high titer, and was referred to as mAb 1F4. Thus, mAb 1F4 was selected for further study. mAb 1F4 was purified from mouse ascites fluid (Fig 2A), and it was characterized by western blotting and an antibodies subclass identification kit. The results showed that mAb 1F4 was belong to the IgG1 subclass and the κ chain (Fig 2B and 2C). Then, we analyzed the specificity of mAb 1F4 by an indirect ELISA, and the results showed that mAb 1F4 recognized only *S*. *aureus* GapC, which suggested that the mAb was generated successfully (Fig 2D). Western blotting also showed that mAb 1F4 recognized only the recombinant *S*. *aureus* GapC protein, even though the sequence homology between *S*. *aureus* and *S*. *dysgalactiae* GapC is 63% (Fig 2E). Because GapC is a surface protein that possesses GAPDH activity and reversibly catalyzes the conversion of glyceraldehyde-3-phosphate into 1, 3-bisphosphoglycerate by phosphorylation, the effect of the mAb on GAPDH activity of the recombinant GapC was determined by GAPDH activity assay kit (OMIN, China). The results showed that mAb 1F4 inhibited the GAPDH activity of *S*. *aureus* GapC (Fig 2F).

To research the protective effect of mAb 1F4, a passive immunoprotection experiment against *S*. *aureus* (Newman) infection was conducted. The results showed that mice in the negative control group died within 20 h after challenge. However, the survival time of mice immunized with mAb 1F4 were 30 h longer than the negative control group (Fig 2G). These results suggest that, although not as effective as GapC anti-serum, mAb 1F4 offered a certain degree of immunoprotection against *S*. *aureus* infection.

### Phage-displayed epitopes biopanned by the mAb

**Fig 3:**
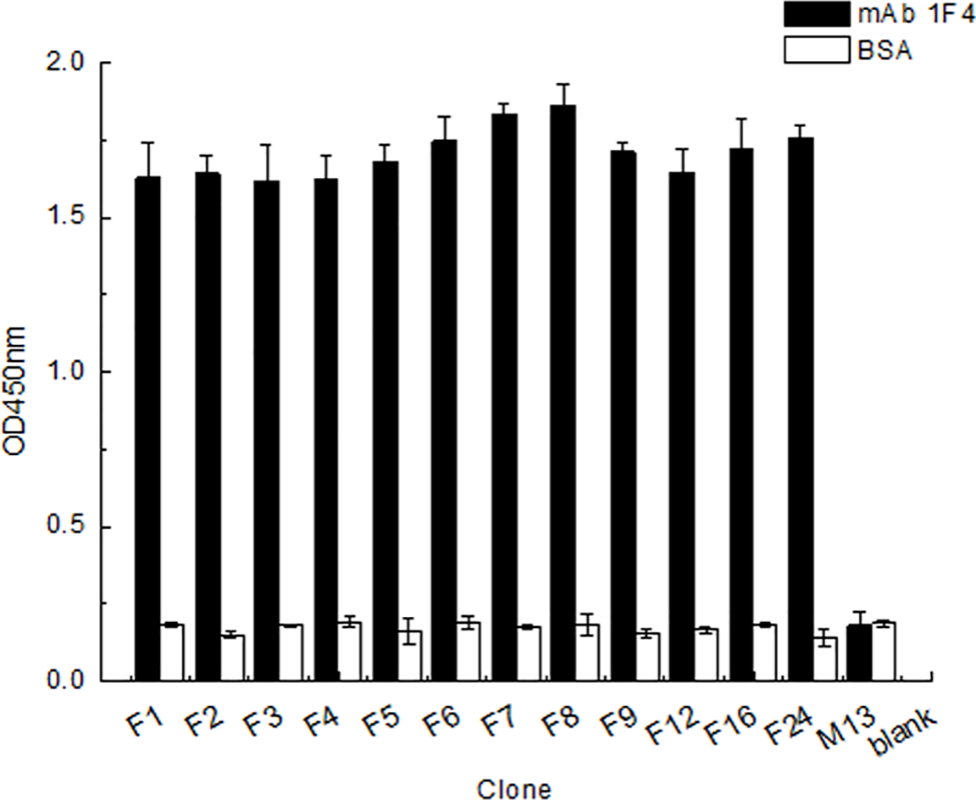
Identification of strongly positive phage clones selected by 1F4 using a sandwich ELISA. The negative control was wild-type M13 phage and the blank control was *E*. *coli* ER2738. BSA-coated wells were used to exclude cross-activity.

The random PhD-12 phage display peptide library was screened with mAb 1F4, and the eluted phage titers were determined by sandwich ELISA after three rounds of biological panning. The phages that were non-specifically bound to mAb 1F4 were removed during the panning. The yield of phages with high specific binding to mAb 1F4 increased continuously with three rounds of panning, proving that the specific phages were enriched (S1 Table). We selected 30 clones for mAb 1F4 and analyzed them by sandwich ELISA. Twelve clones showed strong, positive reactions with mAb 1F4 (Fig 3). We sequenced the positive phages and analyzed the results by aligning the GapC amino acid sequences using DNASTAR software. The amino acid sequences of the 12 clones showed the consensus motif GYTEDEIV (S2 Table). The GYTEDEIV motif is located at amino acids 272–279 of the *S*. *aureus* GapC and it is likely one of the B-cell epitopes of *S*. *aureus* GapC.

### Key amino acids of the epitope

**Fig 4:**
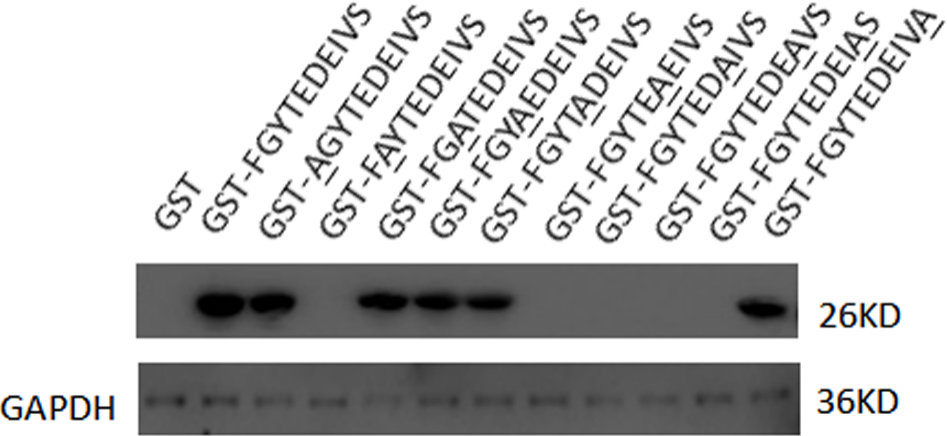
Precisely defining the epitopes. The reactivity of the fusion proteins, in which each amino acid (^271^FGYTEDEIVS^280^) was mutated to alanine, with mAb 1F4 was identified by western blotting. The G272A, D276A, E277A, I278A, and V279A mutations completely disrupted the reactivity of the epitope with mAb 1F4.

Every amino acids of the ^272^GYTEDEIV ^279^ motif was mutated to alanine to verify the epitope exactly (S3 Table). Plasmid pGEX-6P-1 was used to express the mutated *gapC* genes which were GST-fusion protein (S2 Fig). Western blotting was performed with mAb 1F4 as the primary antibody. The results showed that the G272A, D276A, E277A, I278A, and V279A mutations completely disrupted the reactivity of the epitope with mAb 1F4 (Fig 4). The results confirmed that the ^272^GYTEDEIV^279^ motif was indeed the B-cell epitope of the *S*. *aureus* GapC protein, which represents the most basic reactive unit of the epitopes recognized by mAb 1F4.

### The epitope recognized by mAb 1F4 is located on the cell surface

**Fig 5:**
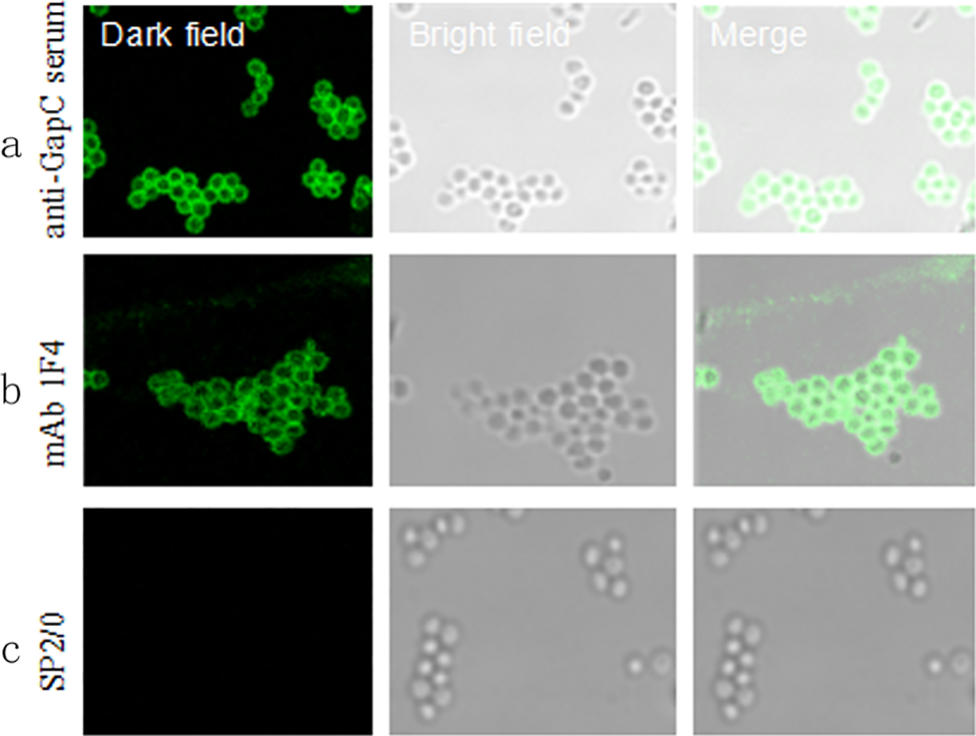
Analysis of the ^272^GYTEDEIV^279^ epitope by confocal laser-scanning microscopy. The left panels show dark field fluorescence photos; the middle panels show bright field photos of the bacteria; and the right panels show the two fields combined. (**a–c**) The *S*. *aureus* (Newman) strain was observed. Primary antibodies were *S*. *aureus* anti-GapC serum, mAb 1F4, and the SP2/0 supernatant, respectively.

To further verify the localization of the ^272^GYTEDEIV^279^ epitope on *S*. *aureus* Newman cells, we used mAb 1F4 as the primary antibody and a FITC goat anti-mouse antibody as the secondary antibody to perform an indirect immunofluorescence assay. The results showed that the mAb 1F4 group showed obvious green fluorescence and the same bacterial morphology was observed in the brightfield (Fig 5). Moreover, the fluorescence was located on the surface of the bacteria, indicating that the ^272^GYTEDEIV^279^ B-cell epitope was exposed on the surface of the bacteria.

### The ^272^GYTEDEIV^279^ epitope is highly conserved

To identify whether the selected epitope was conserved in different *S*. *aureus* strains and other microbial species, the amino acid sequences of the epitope was compared with those of different strains using the BLAST at the NCBI (Table 2). The results of the homology analysis revealed that the ^272^GYTEDEIV^279^ motif is highly conserved in *Staphylococcus spp*.

**Table 2:** Alignment of the sequences surrounding the epitope-coding region of the GapC protein from different *S*. *aureus* strains.

Species	Epitope motif
*Staphylococcus aureus* (AQD18932)	SNESF**GYTEDEIV**SSDVVG
*Staphylococcus aureus* (SGV41094)	SNESF**GYTEDEIV**SSDVVG
*Staphylococcus aureus W52298* (EYG72453)	SNESF**GYTEDEIV**SSDVVG
*Staphylococcus aureus* (SCS75121)	SNESF**GYTEDEIV**SSDVVG
*Staphylococcus aureus* (SHD85683)	SNESF**GYTEDEIV**SSDVVG
*Staphylococcus aureus Chi-8* (EZX77242)	SNESF**GYTEDEIV**SSDVVG
*Staphylococcus aureus H85008* (EWH57997)	SNESF**GYTEDEIV**SSDVVG
*Staphylococcus aureus DAR5841* (EXN96039)	SNESF**GYTEDEIV**SSDVVG
*Staphylococcus aureus subsp*. *aureusUSA300* (EES93581)	SNESF**GYTEDEIV**SSDVVG
*Staphylococcus aureus subsp*. *aureusUSA300* (ABD21603)	SNESF**GYTEDEIV**SSDVVG
*Staphylococcus devriesei* (AGB07469)	SNESF**GYTEDEIV**SSDVVG
*Staphylococcus epidermidis* (AAM28559)	SNESF**GYTEDEIV**SSDVVG
*Staphylococcus epidermidis* (AIR83029)	SNESF**GYTEDEIV**SSDVVG
*Staphylococcus epidermidis* (ACH89386)	SNESF**GYTEDEIV**SSDVVG
*Staphylococcus argenteus* (SGX11541)	SNESF**GYTEDEIV**SSDVVG
*Staphylococcus argenteus* (SGW47323)	SNESF**GYTEDEIV**SSDVVG
*Staphylococcus haemolyticus* (ADF49531)	SNESF**GYTEDEIV**SSDVVG
*Staphylococcus haemolyticus* (KQC20862)	SNESF**GYTEDEIV**SSDVVG
*Staphylococcus gallinarum* (KIR10623)	SNESF**GYTEDEIV**SSDVVG
*Staphylococcus gallinarum* (ABC69184)	SNESF**GYTEDEIV**SSDVVG
*Staphylococcus xylosus* (AAM28566)	SNESF**GYTEDEIV**SSDVVG
*Staphylococcus xylosus* (AID43350)	SNESF**GYTEDEIV**SSDVVG
*Staphylococcus succinus* (ACQ91188)	SNESF**GYTEDEIV**SSDVVG
*Staphylococcus succinus* (ACQ91186)	SNESF**GYTEDEIV**SSDVVG
*Staphylococcus petrasii subsp*. *jettensis* (AEM98035)	SNESF**GYTEDEIV**SSDVVG

Note: The GenBank accession numbers of the used strains are indicated in parentheses. The homologous amino acid residues of the epitope motif are bold.

### anti-epitope serum promotes the phagocytic activity of macrophages

**Fig 6:**
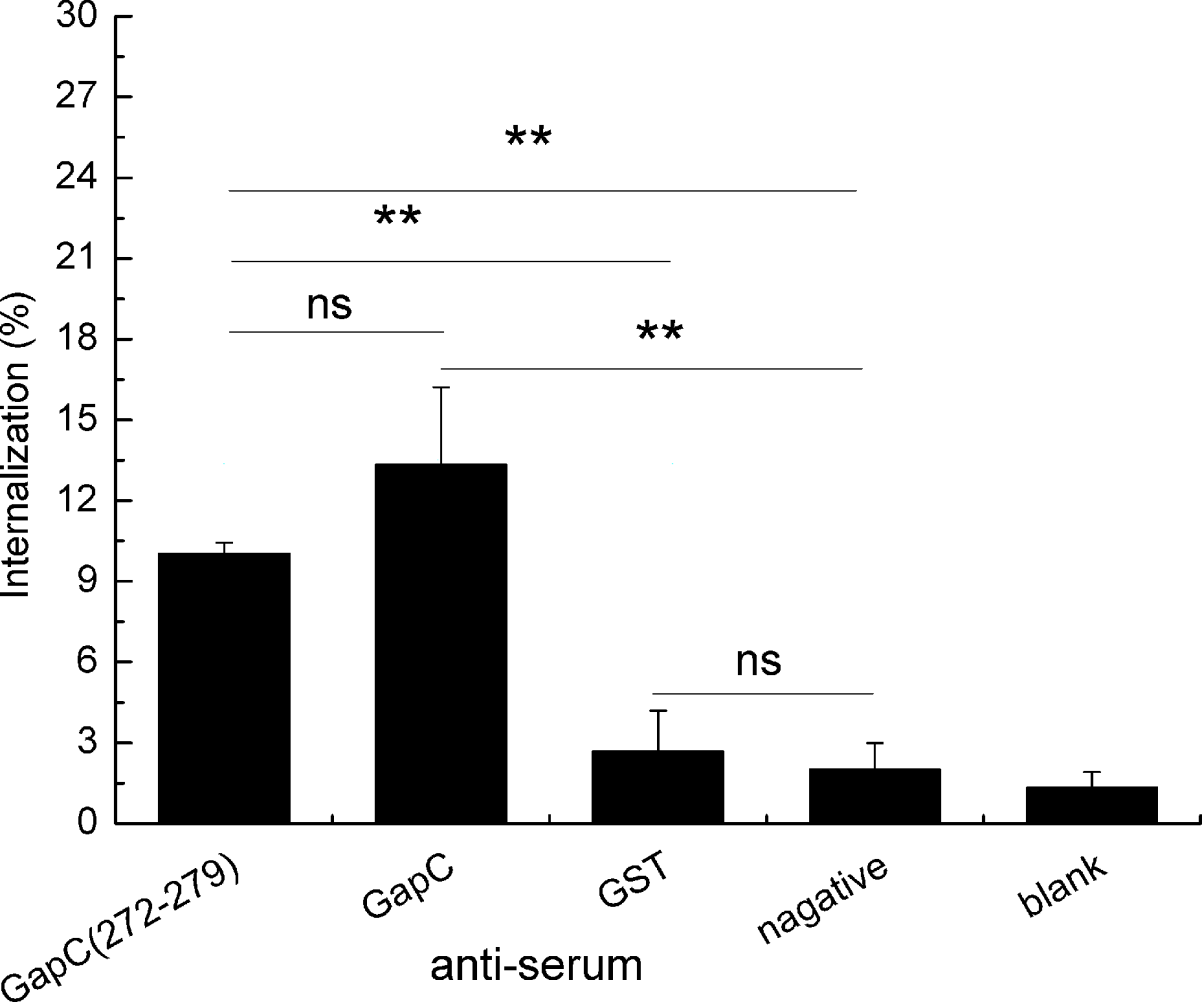
anti-epitope serum-mediated phagocytosis of S. *aureus* Newman. PBS-washed Newman (2×10^5^ CFU) was incubated with 50 μl of the serum for 30 min. The negative control was incubated with the negative serum and the blank control comprised only phagocytes and bacteria. Differences in the internalization rates between anti-epitope peptide serum group and anti-negative serum group was statistically significant. The data represent the means ± SEM (*n* = 3). Statistical significance was measured using a Student’s t-test (**, *p* < 0.01).

To determine whether the anti-epitope serum had the ability to promote macrophage phagocytosis, we incubated *S*. *aureus* with serum. Anti-GapC (272–279) serum was used as the experimental control serum. Anti-GapC serum and negative serum were used as the positive control and negative control serum, respectively. The bacteria were co-cultured with mouse peritoneal macrophages, and the number of phagocytosed bacteria was measured after the cells burst (Fig 6). The results showed that the anti-epitope serum-mediated phagocytosis was significantly higher than that obtained using the GST anti-serum and the negative serum (*p* < 0.01). The internalization rates of the posotive control and anti-epitope prptide serum group were similar. These results suggest that the ^272^GYTEDEIV^279^ B-cell epitope is a functional epitope.

### The ^272^GYTEDEIV^279^ epitope peptide has an immunoprotective against *S*. *aureus* infection

**Fig 7:**
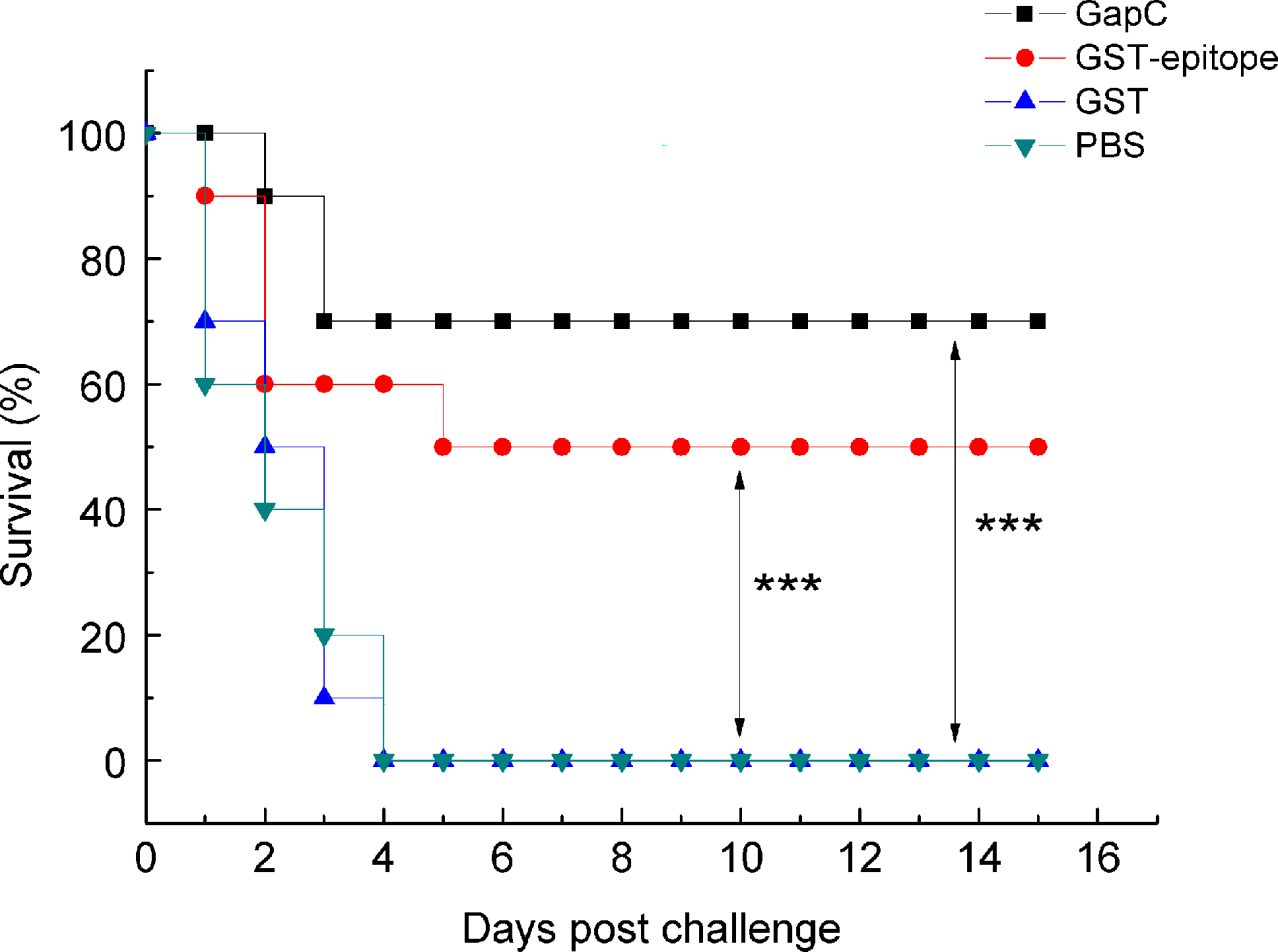
The ^272^GYTEDEIV^279^ epitope peptide confers protection against lethal challenges with *S*. *aureus* Newman. For the prophylaxis study, immunized and control mice (6 weeks old, *n* = 10) were challenged intraperitoneally with *S*. *aureus* (equivalent to 5 × 10^8^ CFU) two weeks after the last immunization. Mice were monitored for 15 days. The survival rates of GapC, GST-epitope were 70% and 50%, respectively. Statistical differences were determined by Student’s t-test. (***, *p* < 0.001).

To detect the ability of the epitope to induce an immune response *in vivo*, we selected 6–8 week-old female BALB/c mice for immunization. An indirect ELISA was used to detect antibody titers of immunized mice 10 days after the third immunization. The results showed that the antibody titer of the GapC protein and the GST-epitope peptide was 1:256,000 and 1:32,000. These results indicated that the B-cell epitope may also induce immune protection. Then, we challenged the mice intraperitoneally with *S*. *aureus*. The recombinant GapC group had the lowest mortality rate of 30%, and the immunoprotection of the ^272^GYTEDEIV^279^ epitope had the second lowest mortality rate of 50%. In contrast, GST and PBS did not induce protective immune responses, and both this two groups of mice died within a short time after lethal challenge (Fig 7). These results suggest that the ^272^GYTEDEIV^279^ B-cell epitope could induce strong immunoprotection against *S*. *aureus* infection, which is consistent with previous results.

### Tertiary structure of the epitope

**Fig 8:**
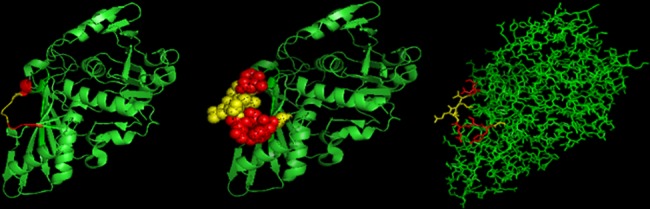
Structural analysis of the linear B-cell epitope. The PDB Protein Data Bank method was used to analyze the three-dimensional model of *S*. *aureus* GapC. The residues of the linear B-cell epitope that are recognized by mAb 1F4 are highlighted in red and shown in cartoon, stick, and dot formats, respectively. The core amino acids are shown in yellow.

Bioinformatics is an efficient way to analyze proteins. Thus, the PDB (http://www.rcsb.org/pdb/home/home.do) method and the PyMOL tool were used to analyze the tertiary structure of GapC and the epitope (Fig 8). The results showed that the epitope ^272^GYTEDEIVSSD^282^ has a coli structure and exposed on the surface of GapC protein.

## Discussion

*Staphylococcus aureus* is rapidly developing resistance to antibiotics, which leads to life-threatening infections, especially in patients whose immune systems have been compromised by other diseases. The prolonged use of antibiotics inhibits antibiotic-sensitive strains and alters the intestinal flora, resulting in *S*. *aureus* infections, for instance, *S*. *aureus* enteritis. Similarly, *S*. *aureus* also causes many medical device-related infections. For animals, *S*. *aureus* also causes various animal infections. Because of the prevalence of these infections and the lack of new antibiotics, immunotherapy for *S*. *aureus* has begun to be studied extensively. Previous studies have shown that traditional inactivated vaccines and capsular polysaccharide vaccines are not ideal for immunoprotection [37, 38]. One reason is that *S*. *aureus* has a variety of capsular types and lacks cross-immune protection between the capsular types. Moreover, the immunogenicity of capsular polysaccharides is weak, which prevents the induction of a strong immune response. In recent years, many studies have focused on subunit vaccines [39, 40]. A variety of antigenic proteins has shown good immunogenicity and can induce organisms to produce an effective immune response. However, because of a plethora of pathogenic factors and the complicated nature of *S*. *aureus* pathogenesis, clinical tests of subunit vaccines produced with a single antigen are still unsatisfactory [41]. Multi-antigen subunit vaccines can achieve significantly improved the immune protection, especially in the presence of adjuvants that can induce cellular immune responses, thereby effectively stimulating a host to produce effective cellular and humoral immune responses. However, the large antigenic molecules and limited expression capacity severely constrains the number of subunits that can be incorporated into multi-antigen subunit vaccines. In contrast, epitope vaccines contain only protective epitopes and are relatively safe, and a more effective immune response can be achieved by connecting several epitopes. Therefore, identifying effective B-cell epitopes of surface antigens is critical for the generation of epitope vaccines against *S*. *aureus*.

Previous studies have shown that GapC, which is located on the surface of the cytomembrane of *S*. *aureus*, is highly conserved and capable of inducing a protective immune response [18, 20]. Here we generated a mAb against *S*. *aureus* GapC. The results showed that mAb 1F4 belonged to the IgG1 subclass and the κ chain. Furthermore, mAb 1F4 only recognized the GapC protein of *S*. *aureus*, and passive immunization with mAb 1F4 significantly increased the survival rate of mice after a *S*. *aureus* challenge, proving that mAb 1F4 could offer protection against *S*. *aureus* infection. To further analyze the minimal unit of the GapC protein that bound to mAb 1F4, we identified the linear B-cell epitope ^272^GYTEDEIVSSD^282^ by screening a phage-displayed random 12-mer peptide library with mAb 1F4. The conservation of the B-cell epitope was demonstrated by aligning the epitope sequences of more than 100 different *Staphylococcus* strains through the BLAST tool at the NCBI, 25 of which were picked randomly and listed in Table 2. The results suggest that the ^272^GYTEDEIVSSD^282^ motif, which is highly conserved and capable of inducing a protective immune response, is a highly conserved epitope on the GapC protein of *Staphylococcus* species. The high degree of conservation provides the possibility for this B-cell epitope to become a potential component of a multi-epitope vaccine, especially to treat *Staphylococcus epidermidis* infections, which have been increasing gradually in recent years. The results of the site-directed mutagenesis analysis showed that mutating the residues G272, D276, E277, I278, and V279 to alanine completely abrogated the reactivity of GapC with mAb 1F4. Therefore, G272, D276, E277, I278, and V279 constitute the core of the ^272^GYTEDEIVSSD^282^ motif.

Analysis of the tertiary structure of the epitope showed that the epitope ^272^GYTEDEIVSSD^282^ has a coli structure that is likely exposed on the surface of the GapC protein. A confocal laser-scanning microscopic analysis demonstrated that this B-cell epitope was recognized by purified mAb 1F4, as green fluorescence showed that the ^272^GYTEDEIVSSD^282^ epitope was exposed on the bacterial surface. At the same time, the B-cell epitope antisera promoted the opsonophagocytic activity of macrophages. Furthermore, positive immunization with the B-cell epitope peptide showed that it could induce effective immunoprotection against a *S*. *aureus* infection. All the above data indicate that the B-cell epitope ^272^GYTEDEIVSSD^282^ is a highly conserved, efficient, and immunodominant epitope, and it has great application potential in the future.

## Conclusions

The data presented here demonstrate that anti-GapC mAb 1F4 might be used in immunotherapeutic treatment of *S*. *aureus* infections, and the B-cell epitope ^272^GYTEDEIVSSD^282^ might be a promising epitope for a multi-epitope vaccine.

## Supporting information

S1 Fig Construction of GapC. The full-length GapC gene was cloned into pET-32a vector; Lane M, Trans5K DNA Marker (Left) and Trans2K DNA Marker (Right); Lane 1, pET-32a-GapC digested with *Bam*H I and *Hind* III; Lane 2, positive clone identified by PCR.
(PDF)

S2 Fig Expression of GST-epitope peptide. (PDF)

S1 Table Enrichment of positive phage clones by biopanning the PhD-12 library. (PDF)

S2 Table Amino acid sequences of PhD-12 phage-displaying peptides from the strongly positive phage clones after bio-panning. Consensus amino acid motifs are shown in bold and underlined.
(PDF)

S3 Table The oligonucleotides encoding the truncated GapC amino-terminus and the alanine-scanning peptides. The mutational sites are shown in lowercase letters. The mutated amino acids are bold and underlined.
(PDF)
