# Supplementary material for: Identification of a protective B-cell epitope of the Staphylococcus aureus GapC protein by screening a phage-displayed random peptide library
Source: PLoS One. 2018 Jan 5;13(1):e0190452. doi: 10.1371/journal.pone.0190452 (PMC5755776; doi:10.1371/journal.pone.0190452)
Supplement: S1 Fig — The full-length GapC gene was cloned into pET-32a vector; Lane M, Trans5K DNA Marker (Left) and Trans2K DNA Marker (Right); Lane 1, pET-32a-GapC digested with BamH I and Hind III; Lane 2, positive clone identified by PCR. (PDF) [file pone.0190452.s001.pdf]

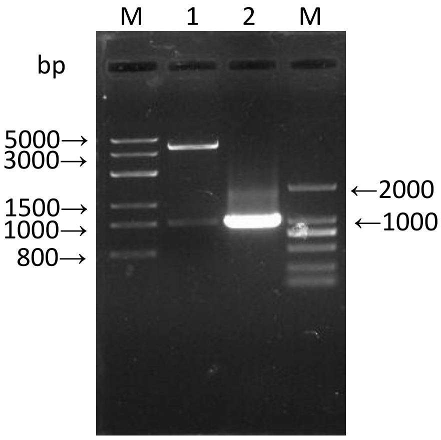

S1 Fig. Construction of GapC. The full-length GapC gene was cloned into pET-32a vector; Lane M, Trans5K DNA Marker (Left) and Trans2K DNA Marker (Right); Lane 1, pET-32a-GapC digested with *Bam*H I and *Hind* III; Lane 2, positive clone identified by PCR.
