# Supplementary figures and images for: Identification of a protective B-cell epitope of the Staphylococcus aureus GapC protein by screening a phage-displayed random peptide library
Source: PLoS One. 2018 Jan 5;13(1):e0190452. doi: 10.1371/journal.pone.0190452 (PMC5755776; doi:10.1371/journal.pone.0190452)

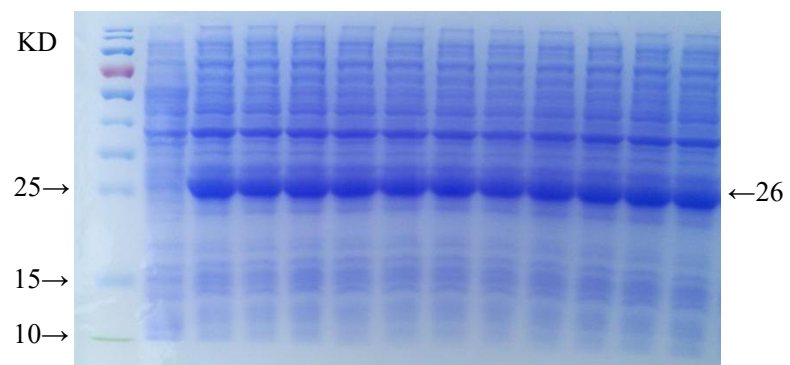

**S2 Fig. Expression of GST-epitope peptide.**

Supplement: S2 Fig — (PDF) [file pone.0190452.s002.pdf]
