# Supplementary material for: Identification of a protective B-cell epitope of the Staphylococcus aureus GapC protein by screening a phage-displayed random peptide library
Source: PLoS One. 2018 Jan 5;13(1):e0190452. doi: 10.1371/journal.pone.0190452 (PMC5755776; doi:10.1371/journal.pone.0190452)
Supplement: S1 Table — (PDF) [file pone.0190452.s003.pdf]

Cycles	mAb 1F4 (mg/L)	Washing (%TBST)	Input (pfu)	Output(pfu)	Yield
1	100	0.1	1.0×10^{11}	2.5×10^{4}	2.5×10^{-7}
2	50	0.3	1.0×10^{11}	6.8×10^{8}	6.8×10^{-3}
3	30	0.5	1.0×10^{11}	7.3×10^{9}	7.3×10^{-2}

S1 Table. Enrichment of positive phage clones by biopanning the PhD-12 library.
