# Supplementary material for: Identification of a protective B-cell epitope of the Staphylococcus aureus GapC protein by screening a phage-displayed random peptide library
Source: PLoS One. 2018 Jan 5;13(1):e0190452. doi: 10.1371/journal.pone.0190452 (PMC5755776; doi:10.1371/journal.pone.0190452)
Supplement: S2 Table — Consensus amino acid motifs are shown in bold and underlined. (PDF) [file pone.0190452.s004.pdf]

Phage	Amino acid sequence of the insert									
1				C	P	**D**	**E**	K	H	Q
2				C	P	**D**	**E**	K	H	Q
3		**G**	L	P	P	R	**E**	**I**	**V**	D
4		K	A	C	P	**D**	**E**	K	H	S
5				**T**	R	N	T	R	T	
6	Q	E	S	Q	**E**	P	D	**I**	H	Y
7		**G**	L	P	P	R	**E**	**I**	**V**	D
8	I	**G**	L	E	M	V	F	Q	L	I
9	I	N	V	**T**	K	L	L	**I**	Q	I
10	Q	E	S	Q	**E**	P	D	**I**	H	Y
11			T	**T**	R	N	T	R	T	R
12	Q	R	N	K	A	A	H	S	**V**	N
Consensus		**G**	Y	**T**	**E**	**D**	**E**	**I**	**V**	
GapC	F	**G**	Y	**T**	**E**	**D**	**E**	**I**	**V**	S

S2 Table. Amino acid sequences of PhD-12 phage-displaying peptides from the strongly positive phage clones after bio-panning. Consensus amino acid motifs are shown in bold and underlined.
