# Supplementary material for: Identification of a protective B-cell epitope of the Staphylococcus aureus GapC protein by screening a phage-displayed random peptide library
Source: PLoS One. 2018 Jan 5;13(1):e0190452. doi: 10.1371/journal.pone.0190452 (PMC5755776; doi:10.1371/journal.pone.0190452)
Supplement: S3 Table — The mutational sites are shown in lowercase letters. The mutated amino acids are bold and underlined. (PDF) [file pone.0190452.s005.pdf]

Coding motifs	Name	The sequences of oligonucleotides
^{271}FGYTEDEIVS^{280}	WT271-281S	5’-gatccTTCGGTTACACTGAAGACGAAATCGTTTCTc-3’
	WT271-281R	5’-tcgagAGAAACGATTTCGTCTTCAGTGTAACCGAAg-3’
^{271}**A**GYTEDEIVS^{280}	F271A-S	5’-gatccGCGGGTTACACTGAAGACGAAATCGTTTCTc-3’
	F271A-R	5’-tcgagAGAAACGATTTCGTCTTCAGTGTAACCCGCg-3’
^{271}F**A**YTEDEIVS^{280}	G272A-S	5’-gatccTTCGCGTACACTGAAGACGAAATCGTTTCTc-3’
	G272A-R	5’-tcgagAGAAACGATTTCGTCTTCAGTGTACGCGAAg-3’
^{271}FG**A**TEDEIVS^{280}	Y273A-S	5’-gatccTTCGGTGCGACTGAAGACGAAATCGTTTCTc-3’
	Y273A-R	5’-tcgagAGAAACGATTTCGTCTTCAGTCGCACCGAAg-3’
^{271}FGY**A**EDEIVS^{280}	T274A-S	5’-gatccTTCGGTTACGCGGAAGACGAAATCGTTTCTc-3’
	T274A-R	5’-tcgagAGAAACGATTTCGTCTTCCGCGTAACCGAAg-3’
^{271}FGYT**A**DEIVS^{280}	E275A-S	5’-gatccTTCGGTTACACTGCGGACGAAATCGTTTCTc-3’
	E275A-R	5’-tcgagAGAAACGATTTCGTCCGCAGTGTAACCGAAg-3’
^{271}FGYTE**A**EIVS^{280}	D276A-S	5’-gatccTTCGGTTACACTGAAGCGGAAATCGTTTCTc-3’
	D276A-R	5’-tcgagAGAAACGATTTCCGCTTCAGTGTAACCGAAg-3’
^{271}FGYTED**A**IVS^{280}	E277A-S	5’-gatccTTCGGTTACACTGAAGACGCGATCGTTTCTc-3’
	E277A-R	5’-tcgagAGAAACGATCGCGTCTTCAGTGTAACCGAAg-3’
^{271}FGYTEDE**A**VS^{280}	I278A-S	5’-gatccTTCGGTTACACTGAAGACGAAGCGGTTTCTc-3’
	I278A-R	5’-tcgagAGAAACCGCTTCGTCTTCAGTGTAACCGAAg-3’
^{271}FGYTEDEI**A**S^{280}	V279A-S	5’-gatccTTCGGTTACACTGAAGACGAAATCGCGTCTc-3’
	V279A-R	5’-tcgagAGACGCGATTTCGTCTTCAGTGTAACCGAAg-3’
^{271}FGYTEDEIV**A**^{280}	S280A-S	5’-gatccTTCGGTTACACTGAAGACGAAATCGTTGCGc-3’
	S280A-R	5’-tcgagCGCAACGATTTCGTCTTCAGTGTAACCGAAg-3’

S3 Table. The oligonucleotides encoding the truncated GapC amino-terminus and the alanine-scanning peptides. The mutational sites are shown in lowercase letters. The mutated amino acids are bold and underlined.
